# Inbreeding load in finite populations from dominant and overdominant mutations

**DOI:** 10.1098/rspb.2025.0845

**Published:** 2025-07-09

**Authors:** Inés González-Castellano, Aurora García-Dorado, Armando Caballero

**Affiliations:** ^1^ Centro de Investigación Mariña, Universidade de Vigo, Vigo, Spain; ^2^ Universidade da Coruña, A Coruña, Spain; ^3^ Universidad Complutense de Madrid, Madrid, Spain

**Keywords:** fitness, heterozygote advantage, balancing selection, inbreeding depression, selfing

## Abstract

Inbreeding depression is a widespread phenomenon that reflects the burden of deleterious effects hidden in heterozygosis in non-inbred populations but exposed in homozygosis in inbred individuals, known as inbreeding load (*B*). This load can be due to partially or fully recessive deleterious mutations (dominance model) or to heterozygote advantage (overdominance model, where both homozygotes are deleterious relative to the heterozygote). There are many studies addressing the changes in inbreeding load in finite populations assuming the dominance model. However, the contribution of overdominance to inbreeding depression has been focused on infinite-size populations. We carried out computer simulations to investigate the joint impact of dominant and pure overdominant mutations on inbreeding load, both for self-fertilizing populations and for panmictic populations suffering from a drastic bottleneck. We found that the overdominant inbreeding load can be substantially reduced by drift even for symmetrical overdominance, at least when considering mutations of small effect. For panmictic bottlenecked populations, the reduction in inbreeding load under dominance and overdominance loci cannot be easily distinguished. However, while purging depletes inbreeding load from dominant loci, slowing inbreeding depression and leading to partial fitness recovery, for overdominant loci fitness declines monotonically.

## 1. Introduction

Inbreeding depression, the observed decline in fitness-related traits in inbred individuals compared to their non-inbred counterparts, is a phenomenon reported in many animal and plant species [1–4] and extensively investigated in numerous studies [5,6]. Inbreeding depression, along with mutational meltdown i.e. the fixation of deleterious mutations by genetic drift [7]), are a major concern for small, endangered populations, as they can lead to extinction [8–12]. In this context, understanding the genetic basis of inbreeding depression is essential to ensure effective conservation management [13–16].

The root cause of inbreeding depression is the increased genomic homozygosity as a consequence of inbreeding. Two main hypotheses can explain why a higher homozygosity lowers fitness [17]. On the one hand, the dominance hypothesis [18] attributes inbreeding depression to the expression of partially or fully recessive deleterious alleles that are hidden in the heterozygotes in large populations but are exposed in homozygosis with inbreeding, lowering the fitness of the populations. On the other, the overdominance hypothesis [19,20] argues that reduced fitness via inbreeding is the result of increased homozygosity at loci with heterozygote advantage (i.e. heterozygotes are superior to both homozygotes). Both hypotheses agree that the origin of inbreeding depression lies in the expression of the inbreeding load [3,17], which can be defined as the expected deleterious effect hidden in heterozygotes per site (which would be expressed in homozygotes by inbreeding), added up over the whole genome. The inbreeding load (*B*), which is expressed in terms of lethal or sterile equivalents [21], is often termed hidden, concealed or masked load [22], as opposed to the expressed load or genetic load (*L*), which is the proportion by which the population fitness is decreased in comparison with an optimum genotype [23,24].

For decades, scientists have debated over the relative importance of dominance and overdominance in inbreeding depression [17,25–30]. Several sources of evidence make it unlikely that overdominance plays a central role in inbreeding depression [11,25,27]. Furthermore, much of the apparent inbreeding depression that was initially thought to be caused by overdominance was later confirmed to be the result of the expression of deleterious recessive mutations at closely linked loci in repulsion (i.e. the so-called pseudo-overdominance [2,28,31–35]). However, true overdominance could also contribute to inbreeding depression. For example, there are a few well-documented cases where overdominance affects fitness-related traits [29,36–38]. Antagonistic pleiotropy can, under some circumstances, produce fitness overdominance in a very natural way [39,40], and there are several sources of evidence suggesting that a fraction of the genome may contain variants with antagonistic pleiotropic effects [41,42]. Thurman & Barrett [43] conducted an extensive meta-analysis of selection coefficients reported in the literature from natural populations of multiple taxa and found that about 2% of the selection coefficients examined could be overdominant.

Under the dominance model, where inbreeding load is only due to (partially) recessive deleterious mutations, the inbreeding load is expected to decline after a population shrinkage because of both genetic drift and purging, the accelerated selection against deleterious mutations as they are expressed in homozygosis by inbreeding (e.g. [44–47]). In fact, purging is prompted by inbreeding either due to reduced population size and/or non-random mating [48]. For panmictic populations of very small size, purging mostly removes recessive deleterious mutations of very large effect, such as semi-lethal and lethal alleles [49,50]. For populations of moderate to large size, however, it can also remove mutations of smaller effect, although its detection may be difficult [51], because of the long time needed for purging to be effective in populations of large size and other issues such as adaptation to captivity or previous purging in endangered populations [52]. Nevertheless, the efficiency of purging has been demonstrated in long-term experiments designed for that purpose [12].

Overdominance represents a form of balancing selection in which alleles can be maintained as polymorphisms at intermediate frequencies in large, outbred populations [53–55]. However, in contrast to the inbreeding load ascribed to recessive deleterious alleles, the load due to overdominant loci cannot be purged in outbred populations, although it is expected to be reduced by genetic drift, particularly in small-size populations, at the cost of lowering population mean fitness [28].

The magnitude of the equilibrium inbreeding depression due to overdominance has also been studied in the context of self-fertilizing species assuming infinite populations [17,55–57]. A relevant feature of this model is that, in the case of symmetrical overdominance (when the two homozygotes have the same disadvantage relative to the heterozygote), the inbreeding depression of selfed progeny relative to outbred progeny (*δ*) is expected to increase with increasing selfing rates. This increase occurs because selfed progeny becomes more inbred the higher the selfing rate, although the inbreeding load *B* remains unchanged. With asymmetrical overdominance, however, inbreeding caused by selfing reduces the protection of the alleles with lower frequency, and, if the selfing rate is sufficiently large, it can put the allele with the lower homozygous fitness at disadvantage, thus producing a sort of purging that favours the less harmful homozygotes. As a consequence, *B* declines with increased selfing, and *δ* increases up to a given selfing rate, but then goes to zero as the allele with highest homozygous fitness becomes fixed [17,55–58]. For other sources of balancing selection, however, *B* may increase or decrease with increasing selfing rates [59].

There are many studies investigating the impact of purging and genetic drift on inbreeding load due to deleterious recessive mutations (the dominance model) for panmictic populations (see, e.g. the review by Dussex *et al.* [47]), and also some studies including partial self-fertilizing populations (e.g. [48,60]). However, there is a scarcity of studies assuming that inbreeding load is contributed both by dominant and overdominant mutations in finite populations. Abu-Awad & Waller [35] recently studied the changes in inbreeding depression in finite populations with partial selfing, considering a pseudo-overdominant genomic region of large effect due to many highly linked partially recessive deleterious alleles. However, similar studies focused on multiple pure overdominant loci of moderately small effects appearing by mutation are lacking. We carried out computer simulations of moderately large populations with increasing rates of self-fertilization, in order to investigate the magnitude of *B* and *δ* expressed by selfed individuals for different selfing rates. Moreover, we simulated panmictic populations subjected to a drastic reduction in size and, therefore, a continued increase in the inbreeding coefficient. We investigated two fitness traits, viability and fecundity, and assumed a dominance model of (partially) recessive deleterious mutations, to which an increasing number of overdominant loci of small effect were added considering two different overdominant patterns. Our results show that increased selfing rates may imply a continuous reduction in both *B* and *δ* of selfed progeny in finite populations, even for a symmetrical model of overdominance, for which *δ* is expected to increase with selfing in infinite populations. For finite panmictic populations, *B* declines with increasing inbreeding under both dominance and overdominance models due to genetic drift, so that the additional decline due to purging of deleterious alleles can be difficult to identify. However, the expected changes in mean fitness under the two scenarios differ substantially.

## 2. Methods

### (a) Fitness models and mutational parameters

Computer simulations were performed using in-house C programs to implement the different fitness models and traits. First, we simulated the genomes of a population consisting of *n* = 1000 diploid individuals (some simulations assumed *n* = 5000). Selfing rates of 0, 25, 50, 75 and 100% were assumed. We followed a mutational model for partially recessive deleterious mutations (dominance model) similar to that considered in other studies [61,62]. Non-recurrent deleterious mutations occurred in free recombining sites at a rate of *U* = 0.2 mutations for the whole haploid genome per generation. A set of simulations was also run assuming a genome length of 20 Morgans, a reasonable genetic length for a variety of species [63], assuming random crossing-overs with no interference. The fitness values assumed were 1, 1 – *sh* and 1 – *s* for the wild-type homozygote, the heterozygote and the mutant homozygote, respectively (table 1), where *s* is the selection coefficient and *h* is the dominance coefficient. The details of the mutational parameters considered for deleterious and lethal mutations are given in the electronic supplementary material, S1.

**Table 1.** Fitness models assumed for a single locus.

locus genotypes	AA	Aa	aa
genotype frequencies	*p* ^2^	2*pq*	*q* ^2^
fitnesses	*W*_AA_	*W*_Aa_	*W*_aa_
dominance model	1	1 – *sh*	1 – *s*
classical overdominance model	1 – *s_o_*	1	1 – *s_o_*
alternative overdominance model	1	1 + *s_o_*	1

Two different overdominance fitness models were considered (table 1). First, the classical model with fitnesses 1 – *s_o_*, 1 and 1 – *s_o_*, where *s_o_* is the selection coefficient against homozygotes. Second, an alternative model with corresponding fitnesses 1, 1 + *s_o_* and 1.

Fitness effects were assumed to be multiplicative across loci, taking into account all mutation types. We considered two fitness traits separately: viability i.e. the probability of survival of offspring) and fecundity (i.e. the number of offspring produced by parents). For the alternative viability model, where the mutant allele causes an increase of the heterozygous fitness, individuals with a viability greater than one were capped to that maximum, as viability is a probability of survival. We assumed a scenario where all mutations conformed to the dominance model (scenario D with only deleterious and lethal or sterile mutations) and a scenario with deleterious and lethal or sterile mutations and also overdominant mutations (D-OD scenario) of moderately small effect. The estimated selection coefficients for overdominant loci (*s_o_*) gathered by Thurman & Barrett [43] are presented in electronic supplementary material, figure S1. These showed a large variation ranging from 0.0007 to 0.88, with a median value of 0.19. Because 29% of the estimates were in the range 0 < *s_o_*≤ 0.05 and 47% in the range 0 < *s_o_*≤ 0.2, we assumed simulated values of 0.02, 0.04 and 0.2. The mutation rates (*U_o_*) assumed were chosen to obtain a sufficiently large number of overdominant loci segregating in the population. Thus, we explored four levels of overdominance: *U_o_* = 0.00005 with *s_o_* = 0.02, *U_o_* = 0.00005 with *s_o_* = 0.04, *U_o_* = 0.0001 with *s_o_* = 0.04 and *U_o_* = 0.00001 with *s_o_* = 0.2. In addition, simulations were performed assuming only overdominant mutations (without deleterious and lethal mutations) in order to confirm the impact of overdominance on some of the results. For most simulations, a classical symmetrical overdominance model was considered. Although this model is improbable [55] their results are the most conservative against loss of polymorphism by genetic drift. Nevertheless, an asymmetrical model (with fitness 1 – *s*_Ao_, 1 and 1 – *s*_ao_) was also considered, for which the fitness effect against one homozygote was twice that against the other homozygote.

The base population was maintained for 10 000 generations under the influence of mutation, recombination, selection and genetic drift under different rates of selfing. Therefore, it should be close to the corresponding equilibrium for scenarios considering only deleterious mutations and should have accumulated a different supply of segregating overdominant loci under the different scenarios including overdominance. Then, a sample of 50 individuals was taken from the base population to estimate the inbreeding load and the inbreeding depression for different selfing rate scenarios, and this process was replicated 200 times. Overdominant mutations fixed in the base population were not considered in these calculations, as these would reduce mean fitness to very low levels. This was not the case for the alternative model, where homozygous genotypes have fitness one. For the panmictic scenario, lines were found using the individuals sampled from the population with no selfing and were maintained for 250 generations with this same size (*n* = 50) and random mating and subjected to the same evolutionary processes and parameters as for the base population. The changes in the mean fitness, inbreeding load and expressed load of the lines were evaluated in each generation and averaged over replicates.

### (b) Total expressed load in panmictic populations

For the panmictic small lines, the total expressed load (*L*), including that due to deleterious and lethal or sterile mutations (mutation load) and that due to overdominant mutations (segregation load; [24]), was obtained in each generation as *L* = (*W*_max_ *– W*) / *W*_max_, where *W* is the mean fitness and *W*_max_ is the maximum fitness value attainable for an individual in each generation, corresponding to the fittest genotype that could be formed from alleles present in that generation. For the classical overdominant model, where overdominant heterozygotes have the maximal fitness of one and the homozygotes have fitness 1 – *s_o_*, *W*_max_ was estimated as *W*_fix_(1 – *s_o_*)^*n*_o_fixed_^*,* where *W*_fix_ is the fitness in the population caused by all deleterious mutations fixed in the lines, and *n*_o_fixed_ is the number of overdominant loci fixed for one or the other allele in the population. This calculation was modified accordingly in the scenario of asymmetrical overdominance, where the homozygotes had different selection coefficients.

For the alternative model for viability and fecundity, for which the fitness of the heterozygote is 1 + *s_o_*, *W*_max_ was estimated as *W*_fix_(1 + *s_o_*)*^no^,* where *n_o_* is the total number of overdominant loci segregating in the lines. That is, *W*_max_ is the maximum possible fitness considering all deleterious mutations fixed in the lines and assuming that all overdominant loci segregating at a given generation are heterozygotes. As mentioned above, in the case of viability, individual viability values (and, therefore, the actual *W*_max_ value) were capped to one in all simulations.

### (c) Inbreeding load and inbreeding depression

The inbreeding load (*B*) was calculated over all segregating selective loci using the expression *B* = Σ2*dpq* [21], where *p* and *q* are the frequencies of the wild and mutant allele, *d* is the difference between the heterozygous and the mid-homozygous values and the summation is for all segregating loci. For deleterious and lethal alleles, *d* = *s*(*h –* 1/2) (e.g. [64, p. 47]). For a symmetrical model of overdominance, where the two homozygotes have the same disadvantage (*s_o_*), *d* = *s*, where *s* = *s_o_* for the classical model and *s* = *s_o_* / (1 + *s_o_*) for the alternative model. The expected inbreeding load from a single symmetric overdominant locus is *B_o_* = *s_o_*/2 regardless of the level of inbreeding (electronic supplementary material, S1) and for *n*_o_ loci with the same effect, *B*_o_ = *n*_o_× (*s_o_*/2).

Inbreeding depression is the reduction of fitness in inbred individuals or in the average fitness of generations where inbreeding has accumulated (inbred mean fitness *W_i_*), compared to that of a reference non-inbred population (outbred mean fitness *W_o_*). The inbred mean fitness depends on the inbreeding coefficient, and, if purging is ignored, can be predicted as


(2.1) $$W_{i}=W_{o}exp(–BF_{i})$$

[21], where *F_i_* is the inbreeding coefficient of inbred individuals. According to this expression, after a reduction in size of a panmictic population, the expected fitness progressively declines at a rate *B* as inbreeding progresses. In the absence of purging and under simplified conditions, the inbreeding load *B* equals the rate of inbreeding depression (δ*). In practice, however, genetic purging will reduce the frequency of deleterious alleles implying smaller fitness declines [46].

In (partially) self-fertilizing populations, the term inbreeding depression, however, is often used to describe the reduction expected in the fitness of selfed progeny (*W_s_*) relative to that of outbred progeny of unrelated parents (*W_o_*) and is defined in the log scale as


(2.2) $$\delta =ln(W_{o}/W_{s})=BF_{s},$$

where *F_s_* is the inbreeding coefficient of selfed progeny. In panmictic populations, the expected value for *δ* is *B*/2. For non-panmictic scenarios, such as for partial self-fertilizing populations, *δ* is expected to increase linearly with the average inbreeding coefficient (*F*) of the population [17,55,58]. Thus, for an infinite population with a proportion *S* of self-fertilization, where the equilibrium inbreeding coefficient is *F* = *S* / (2 – *S*) [64, p. 94], the inbreeding of the offspring obtained by selfing is *F_s_* = (1/2)(1 + *F*) = 1 / (2 – *S*) and, from equation (2.2), the expected inbreeding depression in selfed progeny is *δ* = (*B* / 2)(1 + *F*) = *B* / (2 – *S*) [17]. The inbreeding load *B* can be estimated from equation (2.1) as the rate of inbreeding depression, which can be approximated by δ* = ln(*W_o_*/*W_s_*)/(*F_s_ – F_o_*), where *F_s_* and *F_o_* are the inbreeding of the selfed and non-selfed progeny, respectively, the latter due to background relatedness. To evaluate this estimate of the inbreeding load, the outbred progeny was obtained by establishing pairs of randomly chosen individuals as parents to produce outbred progeny. Additionally, individuals of the population were also self-fertilized to generate selfed progeny. The inbreeding coefficients of individuals were estimated by the estimator proposed by Yang *et al.* [65], considering the simulated neutral loci,

$\hat{F}=1/L\sum_{k=1}^{L}\left[\left(x_{k}^{2}-\left(1+2p_{k}\right)x_{k}+2p_{k}^{2}\right)/\left(2p_{k}\left(1-p_{k}\right)\right)\right]$, where *L* is the total number of loci, $x_{k}$ is the number of minor alleles of locus *k* (i.e. 0, 1 or 2 copies) and $p_{k}$ is the frequency of the minor allele.

## 3. Results

We first address the changes in inbreeding load and inbreeding depression in large populations under partial selfing and, thereafter, those in panmictic populations subjected to a drastic bottleneck.

### (a) Self-fertilizing populations

**Figure 1.**
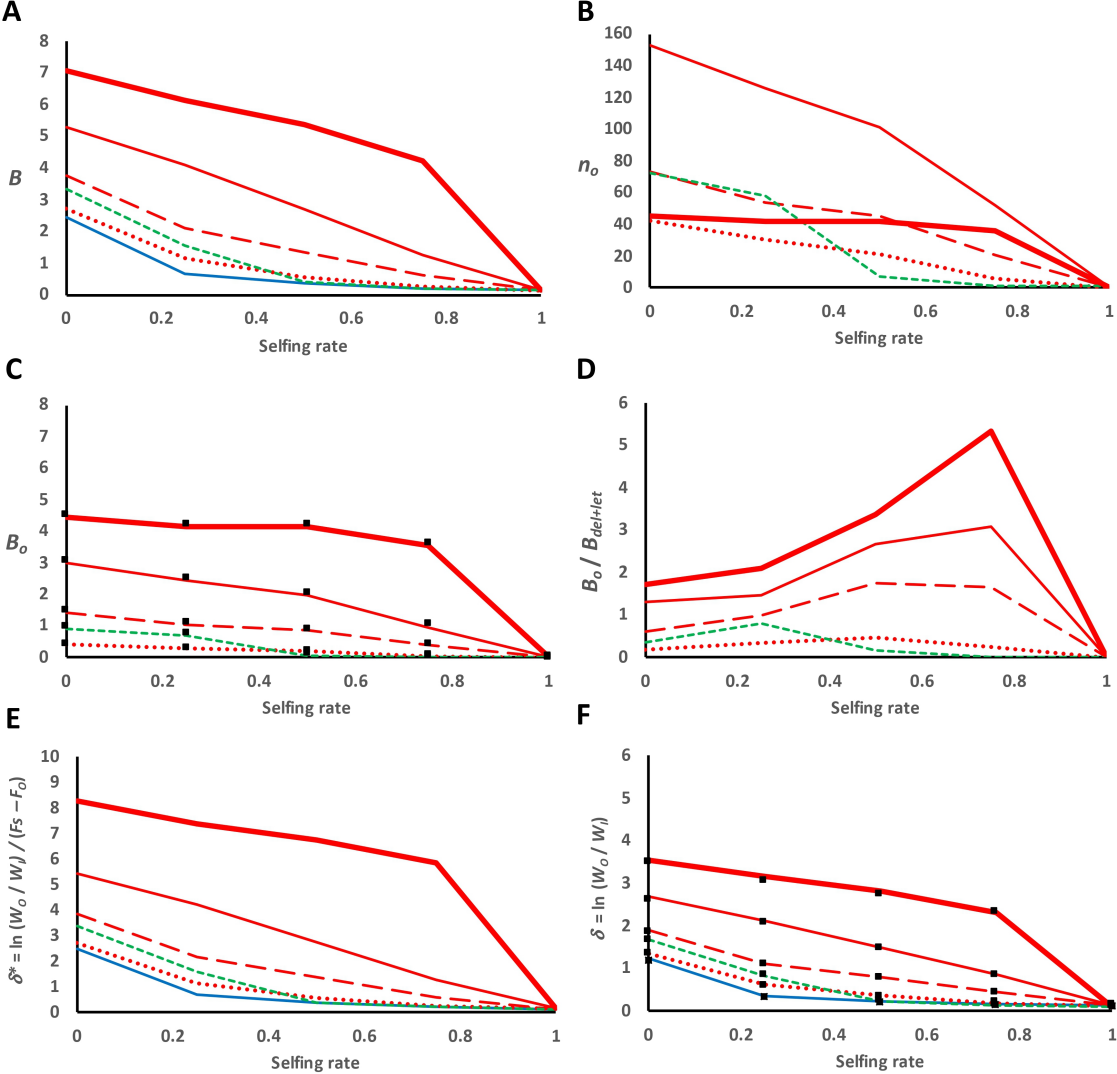
Classical overdominance model for viability in partially selfed populations. Fitness values 1 – *s*_Ao_, 1 and 1 – *s*_ao_ for genotypes AA, Aa and aa, respectively, are assumed for a population of *n* = 1000 individuals subjected to different rates of self-fertilization. Expected total inbreeding load from all selective loci (*B*; panel A), number of overdominant loci segregating in the population (*n_o_*; panel B), expected inbreeding load from overdominant loci (*B_o_*; panel C), ratio between the inbreeding load due to overdominant loci (*B_o_*) and that due to deleterious and lethal mutations (*B*_del + let_) (panel D), rate of inbreeding depression (δ*; panel E) and inbreeding depression of selfed progeny (*δ*; panel F). Black squares represent expected values (see formulae in text). Blue solid lines refer to a dominance model (scenario D; only deleterious and lethal mutations). Red lines indicate the addition of symmetrical overdominant mutations (scenario D-OD) with different mutation rates (*U*_o_) and effects (*s*_Ao_ = *s*_ao_ = *s*_o_): *U*_o_ = 0.00005 and *s_o_* = 0.02 (dotted lines); *U*_o_ = 0.00005 and *s_o_* = 0.04 (broken lines); *U*_o_ = 0.0001 and *s_o_* = 0.04 (thin solid lines); *U*_o_ = 0.00001 and *s_o_* = 0.2 (thick solid lines). The green dashed lines denote the asymmetrical overdominance model: *U*_o_ = 0.0001 and *s*_Ao_ = 0.04, *s*_ao_ = 0.02.

Figure 1 shows results corresponding to the different D and D-OD scenarios for a classical (symmetrical or asymmetrical) model of overdominance considering viability as the fitness trait. The inbreeding load *B* (figure 1A) and its estimate δ* (figure 1E), as well as *δ* (figure 1F), decreased for increasing selfing rates both for a model assuming only deleterious and lethal mutations (D scenario; blue line), or also overdominant mutations (D-OD scenario; red lines), and this decline was faster for an asymmetrical model of overdominance (dashed green lines), as expected. The reduction of inbreeding load ascribed to overdominant loci (figure 1C) was due to the decrease in the number of overdominant segregating loci with increasing selfing rates (figure 1B). However, for the largest overdominant effect considered (*s_o_* = 0.2), the loss of overdominant loci only occurred for the highest selfing rate (figure 1B), and *B_o_* was therefore held almost invariable until that full selfing rate (figure 1C). This shows that, for sufficiently large overdominant effects, the effect of drift can become negligible even for large levels of selfing, as deduced for infinite-size populations. The ratio between the inbreeding load from OD and D mutations (*B_o_*/*B*_del + let_) increased for increasing selfing rates to drop to zero with a very high selfing rate (figure 1D). As the selfing rate is increased, the inbreeding load from deleterious and lethal mutations is lost by drift and purging faster than the loss of *B_o_*, thus explaining the increase in their ratio. For fully selfed populations, no overdominant mutations are kept, and the ratio goes to zero.

The black squares in the figures indicate the expected values given in the previous section (with the corresponding adjustment in the case of an asymmetrical model of overdominance). For example, for the symmetrical model, E[*B_o_*] = *n_o_*× *s_o_*/2, where *n_o_* is the observed number of overdominant loci (figure 1B), and the expected inbreeding depression is *δ* = (*B* / 2) (1 + *F*) (black squares in figure 1F), where *δ* is expected to increase with the selfing rate in infinite populations due to increased *F*, but actually decreases in finite populations because of the decline in *B*.

The simulations presented in figure 1 were also carried out for other models and scenarios. First, a larger population with size *n* = 5000 individuals rather than 1000 was considered, and the results are shown in electronic supplementary material, figure S2. Second, simulations assumed a genome length of 20 Morgan rather than free recombination, with results presented in electronic supplementary material, figure S3. Third, the simulations of figure 1 were repeated by assuming an alternative model for fecundity (electronic supplementary material, figure S4). The results of these cases were very similar, qualitatively, to those given in figure 1. Finally, simulations were run where only overdominant mutations were allowed, without partially recessive deleterious mutations, showing results coherent with those of figure 1 (electronic supplementary material, figure S5).

### (b) Panmictic populations after a bottleneck: classical overdominance model

**Figure 2.**
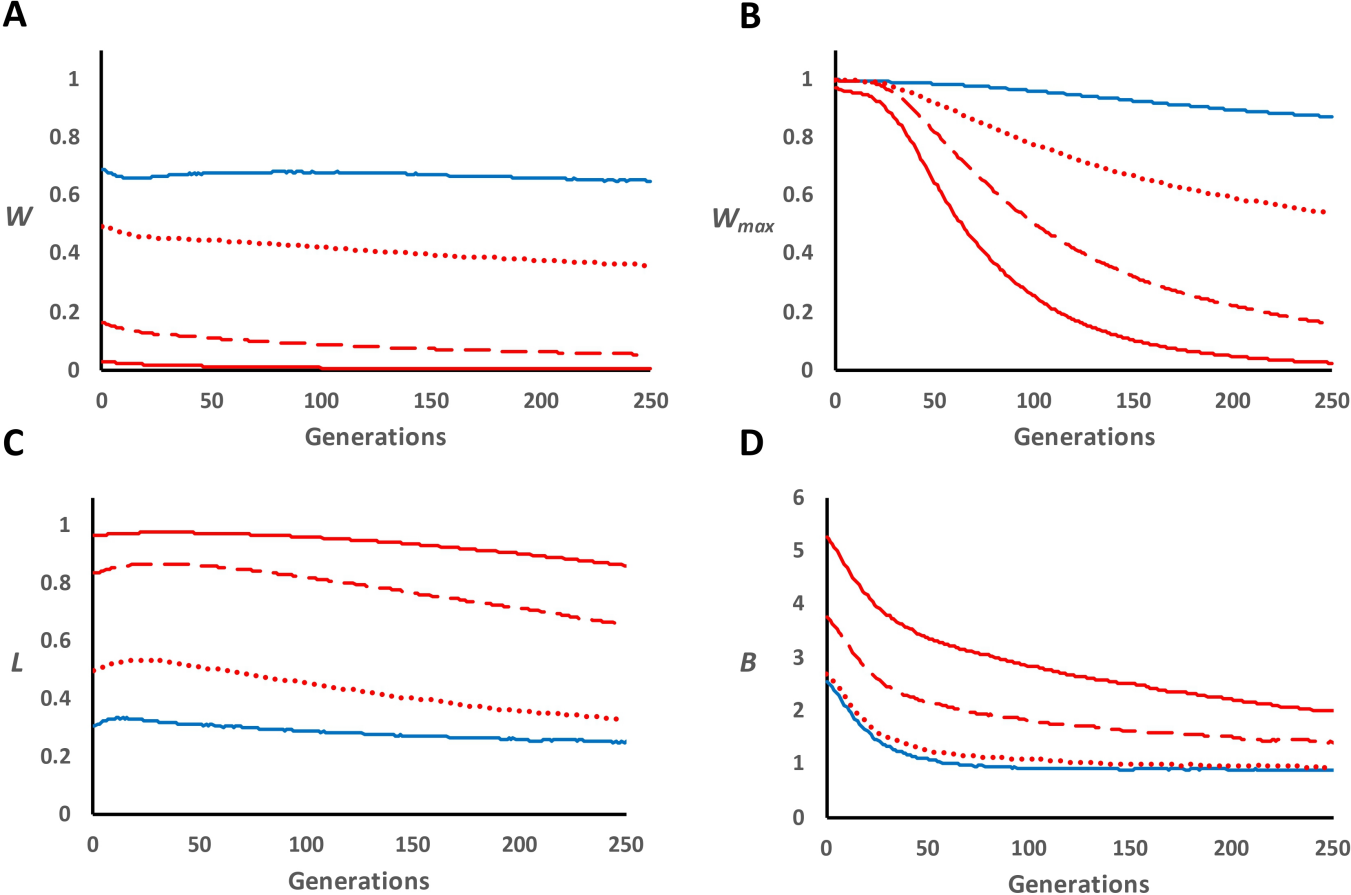
Classical overdominance model for viability in panmictic populations subjected to a drastic bottleneck. Fitness values 1 – *s_o_*, 1 and 1 – *s_o_* are assumed for genotypes AA, Aa and aa, respectively. Evolution over generations of average fitness (*W*; panel A), maximum fitness (*W*_max_; panel B), total expressed load (*L*; panel C) and expected inbreeding load (*B*; panel D), of lines with *n* = 50 individuals after a population bottleneck (generation 0) from a large base panmictic population (*n* = 1000). Blue solid lines refer to a dominance model (scenario D; only deleterious and lethal mutations), while red lines indicate the addition of overdominant mutations (scenario D-OD) with different mutation rates (*U*_o_) and effects (*s*_o_): *U*_o_ = 0.00005 and *s*_o_ = 0.02 (dotted lines); *U*_o_ = 0.00005 and *s*_o_ = 0.04 (broken lines); *U*_o_ = 0.0001 and *s*_o_ = 0.04 (thin solid lines).

Figure 2 shows the change in different parameters over generations for panmictic lines of small population size (*n* = 50) derived from a large population, and a partition of results regarding the different types of mutations is shown in table 2. For the D scenario, the average fitness (*W*) declined initially due to inbreeding depression with a later almost complete recovery, up to 98.8% of the initial value after 100 generations, and a later very slow decay due to continuous deleterious mutation. In contrast, *W* was initially much lower for scenarios involving overdominant mutations (figure 2A), as expected from the Mendelian segregation of overdominant loci, and declined monotonically down to 85% of the initial average after 100 generations for the less intense overdominance scenario (*U_o_* = 0.00005, *s_o_* = 0.02) and down to just 30% of the initial average for the most severe overdominance one (*U_o_* = 0.0001, *s_o_* = 0.04; see last column of table 2). The maximal fitness (*W*_max_) was reduced over time due to the loss or fixation of overdominant alleles (figure 2B), and the total expressed load (*L*; including deleterious and lethal mutation loads and overdominant segregation load) was very large during the whole process and close to one for the scenario with more overdominant loci of large effect (figure 2C).

**Table 2.** Simulation statistics for a classical overdominance model for viability with fitnesses 1 – *s_o_*, 1 and 1 – *s_o_* for genotypes AA, Aa and aa, respectively, assuming absence of overdominant mutations (*U_o_* = 0) or occurrence of overdominant mutations appearing with a rate *U_o_* and effect *s_o_*. Average number of deleterious (*n*_del_; mutations with *s* < 0.9), lethal (*n*_let_; mutations with *s* ≥ 0.9) and overdominant (*n_o_*) mutations segregating in the lines at different generations (*t*), and their average allele frequency (*q*). *B_o_* is the contribution of overdominant mutations to inbreeding load. The last column shows the proportional decline in fitness relative to that at generation 0.

*t*	deleterious			lethals			overdominant			fitness decline
	*n*_del_	*q*	*B*_del_	*n*_let_	*q*	*B*_let_	*n_o_*	*q*	*B_o_*	Δ*W* (%)
***U_o_* = 0**										
0	1486.0	0.09	1.64	73.8	0.01	0.90	0.0			0.0
50	385.6	0.28	0.72	11.5	0.04	0.38	0.0			1.7
100	278.5	0.29	0.57	10.8	0.04	0.35	0.0			1.3
150	222.1	0.27	0.56	10.7	0.03	0.34	0.0			2.2
200	187.2	0.23	0.55	10.9	0.04	0.35	0.0			4.0
250	167.2	0.20	0.54	11.1	0.04	0.36	0.0			5.4
***U_o_* = 0.0001, *s_o_* = 0.04**										
0	1549.9	0.09	1.46	66.3	0.01	0.82	153.4	0.50	3.00	0.0
50	404.3	0.28	0.72	11.1	0.04	0.37	144.0	0.50	2.29	52.6
100	289.2	0.29	0.60	10.7	0.04	0.34	122.1	0.50	1.90	69.9
150	229.8	0.27	0.58	11.2	0.04	0.36	101.3	0.50	1.57	79.4
200	194.0	0.24	0.57	10.8	0.03	0.34	84.2	0.50	1.31	85.0
250	170.2	0.21	0.56	11.1	0.04	0.37	69.8	0.50	1.09	88.6

The inbreeding load (*B*; figure 2D) was initially much larger with overdominant mutations, as expected, and decreased across time in all cases. For the D scenario, *B* dropped much more than expected from drift alone during about 50 generations (by a 51% and 62% factor for the non-lethal and the lethal *B* fractions, respectively) due to genetic purging of deleterious and lethal alleles, and then approached an equilibrium value corresponding to the new mutation-selection-drift balance. As expected [49,50], the removal of lethal mutations occurred faster (in the first period of 50 generations) than for deleterious mutations, which occurred continuously over the whole period of 250 generations (table 2). For scenarios including overdominant alleles, the initial reduction of the overdominant fraction of *B* was slower (by a 24–30% factor), so that the ratio of the inbreeding load arising from overdominant loci to that arising from deleterious and lethal mutations (D scenario) increased over generations up to generation 50 or so (electronic supplementary material, figure S6A), as observed for increased partial selfing in the previous section. After the non-overdominant fraction of *B* reached the new mutation-selection-drift equilibrium, the overall *B* still showed a slow but continuous drop due to the loss or fixation of overdominant loci caused by genetic drift (table 2 and electronic supplementary material, figure S7). For example, the average number of segregating overdominant mutations in the most extreme overdominant case was 153 at generation 0 and decreased to 70 at generation 250 (table 2), and the variance of their frequencies increased with generations (electronic supplementary material, figure S7). The proportional decay in the number of deleterious (*n*_del_) and lethal (*n*_let_) mutations remained about the same regardless of the absence or presence of overdominant loci. However, the average frequency of deleterious mutations was higher in the presence of overdominance mutations in the later generations (table 2).

Considering an asymmetrical model of overdominance in which the selection coefficient for one homozygote is twice as large as that for the other homozygote showed results very close to those for the symmetrical model (electronic supplementary material, figure S8). Similar results to those of figure 2 were also obtained for a classical fecundity model (electronic supplementary material, table S1 and figure S9).

### (c) Panmictic populations after a bottleneck: alternative overdominance model

**Figure 3.**
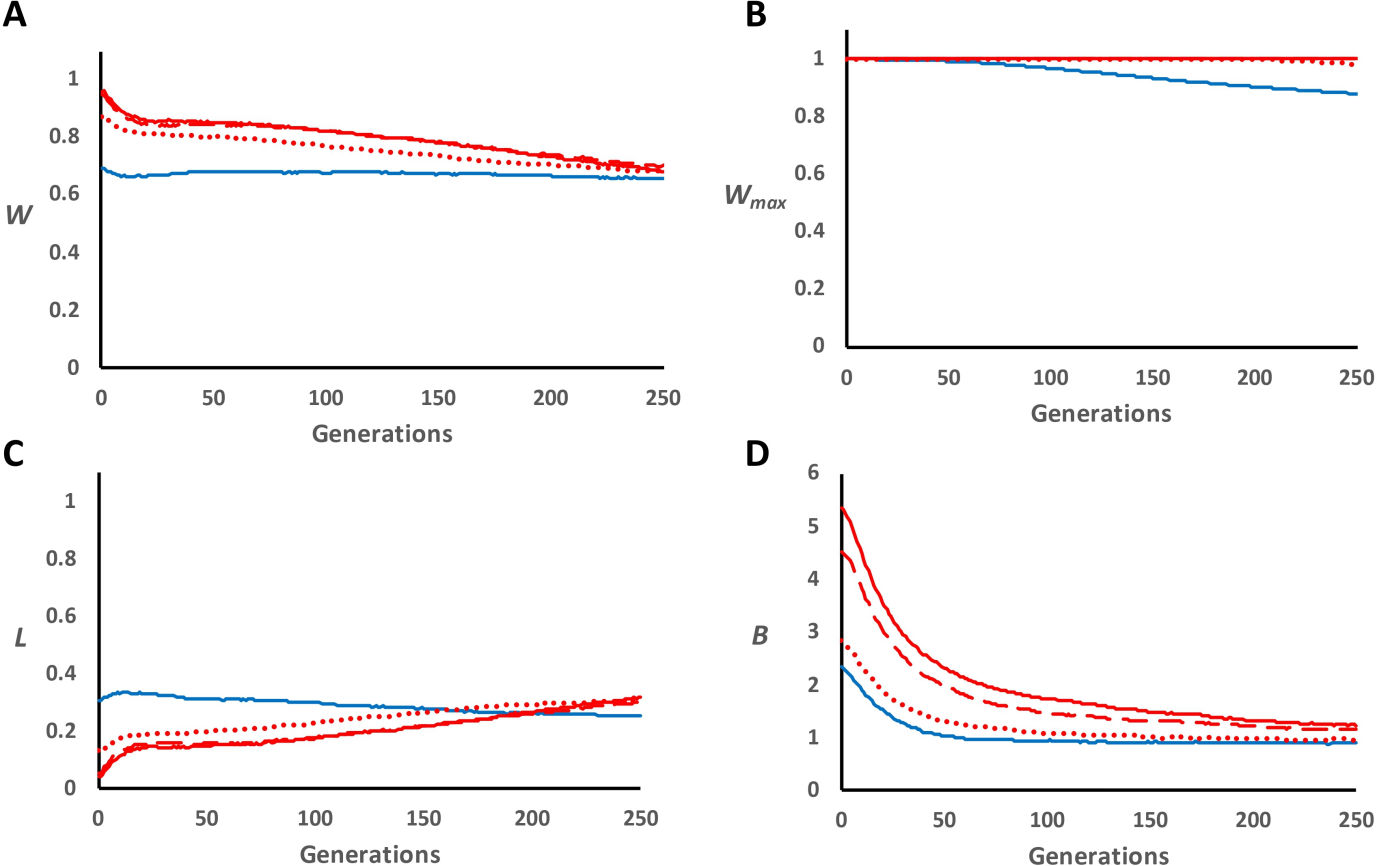
Alternative overdominance model for viability in panmictic populations subjected to a drastic bottleneck. Fitness values 1, 1 + *s_o_* and 1 are assumed for genotypes AA, Aa and aa, respectively. Evolution over generations of average fitness (*W*; panel A), maximum fitness (*W*_max_; panel B), total expressed load (*L*; panel C) and expected inbreeding load (*B*; panel D), of lines with *n* = 50 individuals after a population bottleneck (generation 0) from a large base panmictic population (*n* = 1000). Blue solid lines refer to a dominance model (scenario D; only deleterious and lethal mutations), while red lines indicate the addition of overdominant mutations (scenario D-OD) with different mutation rates (*U*_o_) and effects (*s*_o_): *U*_o_ = 0.00005 and *s*_o_ = 0.02 (dotted lines); *U*_o_ = 0.00005 and *s*_o_ = 0.04 (broken lines); *U*_o_ = 0.0001 and *s*_o_ = 0.04 (solid lines).

The assumption of an overdominance model where the fitness of the two homozygotes is one and that of the heterozygote is 1 + *s_o_* provided viability results rather different from those of the classical model (figure 3 and table 3). For this alternative model, the overdominant loci increase viability in the heterozygotes, but this cannot be higher than one and must be capped at this value. Therefore, the average viability was close to one initially and decreased slowly in a continuous way (figure 3A), the maximal fitness was always close to 1 (figure 3B) and the total expressed load increased continuously as the mean declined (figure 3C). Despite the abovementioned large differences between this model and the classical one in mean fitness and expressed load, the values of the inbreeding load (*B*) (figure 3D) evolved very similarly as those for the classical model, although with a steeper decline for the alternative model (cf. figure 2D). Note that, because this model implies almost no decrease in fitness, the expressed load (*L*) is kept low, and there is a relaxation of selection for deleterious mutations (electronic supplementary material, S1). Moreover, the segregation of dominant and overdominant loci implies a substantial latent inbreeding load (*B*). The ratio of *B* from overdominant loci to that from dominant loci increased over generations (electronic supplementary material, figure S6B), in similitude with the classical model.

**Table 3.** Simulation statistics for an alternative overdominance model for viability with fitnesses 1, 1 + *s_o_* and 1 for genotypes AA, Aa and aa, respectively, assuming absence of overdominant mutations (*U_o_* = 0) or occurrence of overdominant mutations appearing with a rate *U_o_* and effect *s_o_*. Average number of deleterious (*n*_del_; mutations with *s* < 0.9), lethal (*n*_let_; mutations with *s* ≥ 0.9) and overdominant (*n_o_*) mutations segregating in the lines at different generations (*t*), and their average allele frequency (*q*). *B_o_* is the contribution of overdominant mutations to inbreeding load. The last column shows the proportional decline in fitness relative to that at generation 0.

*t*	deleterious			lethals			overdominant			fitness decline
	*n*_del_	*q*	*B*_del_	*n*_let_	*q*	*B*_let_	*n_o_*	*q*	*B_o_*	Δ*W* (%)
***U_o_* = 0**										
0	1509.1	0.09	1.51	65.8	0.01	0.83	0.0			0.0
50	393.3	0.28	0.69	11.0	0.04	0.36	0.0			1.8
100	282.9	0.29	0.60	11.0	0.04	0.36	0.0			2.3
150	224.7	0.27	0.57	11.1	0.04	0.36	0.0			2.9
200	189.5	0.23	0.56	11.4	0.03	0.36	0.0			4.0
250	168.5	0.20	0.55	10.8	0.04	0.35	0.0			5.2
***U_o_* = 0.0001, *s_o_* = 0.04**										
0	2059.3	0.10	2.51	116.5	0.02	1.67	66.8	0.47	1.16	0.0
50	548.2	0.30	1.07	12.5	0.04	0.43	57.2	0.49	0.82	11.0
100	369.1	0.32	0.74	11.6	0.04	0.37	44.0	0.50	0.63	13.9
150	275.3	0.30	0.64	10.8	0.04	0.35	34.8	0.50	0.51	18.1
200	219.8	0.26	0.58	10.7	0.03	0.33	27.9	0.50	0.41	23.4
250	186.1	0.23	0.55	11.0	0.04	0.35	22.8	0.50	0.34	28.6

**Figure 4.**
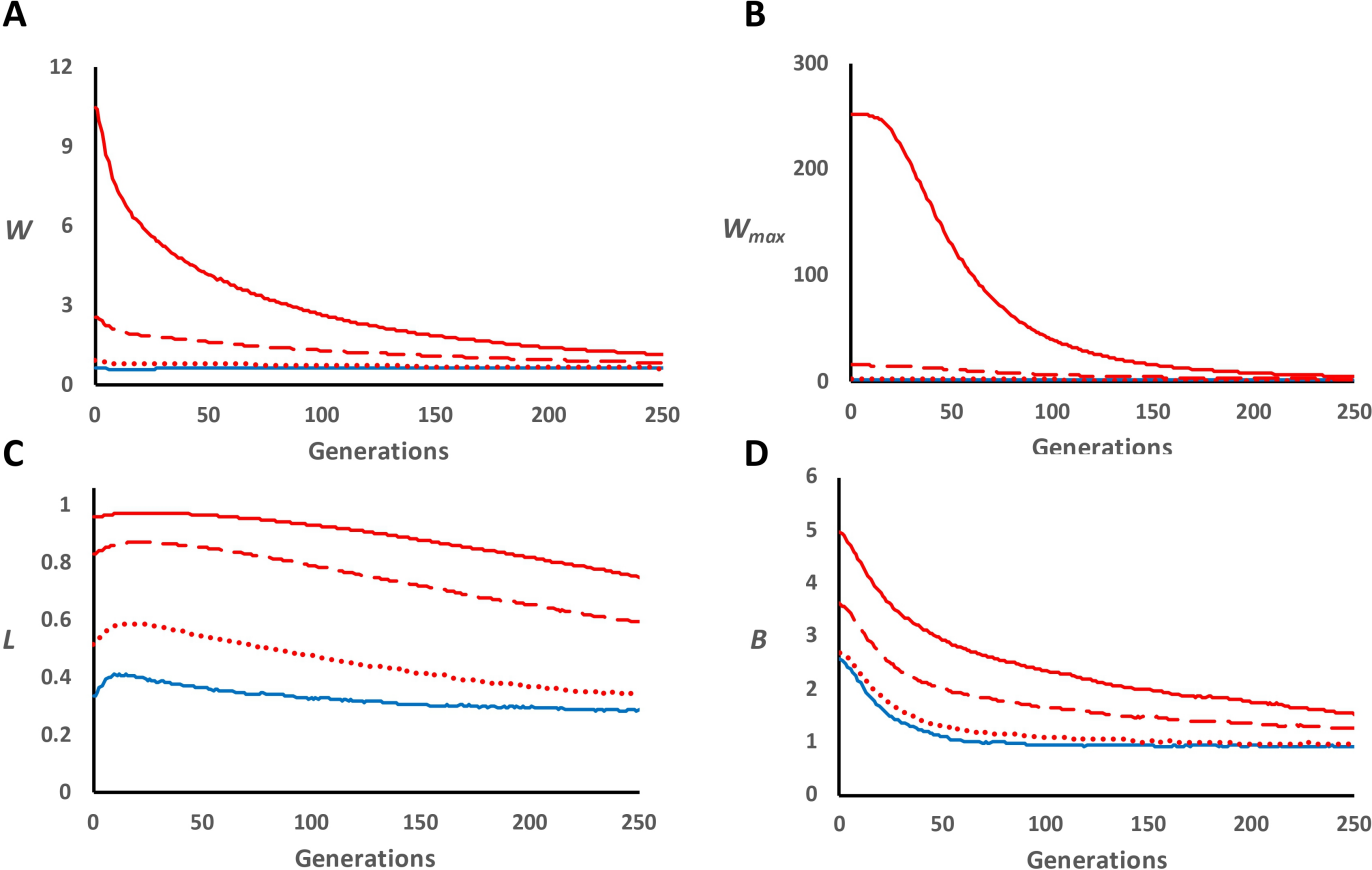
Alternative overdominance model for fecundity in panmictic populations subjected to a drastic bottleneck. Fitness values 1, 1 + *s_o_* and 1 are assumed for genotypes AA, Aa and aa, respectively. Evolution over generations of average fitness (*W*; panel A), maximum fitness (*W*_max_; panel B), total expressed load (*L*; panel C) and expected inbreeding load (*B*; panel D), of lines with *n* = 50 individuals after a population bottleneck (generation 0) from a large base panmictic population (*n* = 1000). Blue solid lines refer to a dominance model (scenario D; only deleterious and sterile mutations), while red lines indicate the addition of overdominant mutations (scenario D-OD) with different mutation rates (*U*_o_) and effects (*s*_o_): *U*_o_ = 0.00005 and *s*_o_ = 0.02 (dotted lines); *U*_o_ = 0.00005 and *s*_o_ = 0.04 (broken lines); *U*_o_ = 0.0001 and *s*_o_ = 0.04 (solid lines).

The abovementioned results refer to the alternative viability model. The alternative model for fecundity gave results very similar to those for the classical overdominance model (viability or fecundity; figure 4 and electronic supplementary material, table S1 and figure S6C). Of course, the mean fecundity and the maximum fecundity (figure 4A,B) were much larger than one in this scenario, but the resulting total expressed load (*L*) and inbreeding load (*B*) (figure 4C,D) were very close to those of the classical model (figure 2C,D).

## 4. Discussion

### (a) Changes in the inbreeding load of self-fertilizing populations

Theoretical studies for a symmetrical model of overdominant effects in an infinite equilibrium population show that the inbreeding depression of selfed progeny relative to outbred individuals (*δ*) is expected to increase as the selfing rate *S* of the population increases [17,55–58]. This occurs despite the equilibrium inbreeding load (*B*) being invariant to *S*, as *δ* = *B* / (2 – *S*) irrespective of the gene action (i.e. both for overdominant and dominant loci; see [17]). For asymmetrical models of overdominance, the inbreeding depression of selfed progeny *δ* increases slightly with *S* until a critical selfing rate and then declines due to the progressive reduction of *B* [55–59].

Overdominance generates a substantial amount of inbreeding load in comparison with that produced by recessive mutations, and the relative difference between these two loads increases for increasing inbreeding values (figure 1; electronic supplementary material, S2−S4). In finite populations, overdominant alleles of large effect (e.g. *s_o_* = 0.2) can be maintained for a large range of selfing rates (figure 1B). However, those of small effect are lost by genetic drift, implying a decrease of both *B* and *δ* with increasing selfing rates. The strength with which overdominant selection protects polymorphisms by favouring the allele with the lower frequency is reduced by increasing selfing because, the larger *S*, the more unlikely is that a gamete carrying that allele will produce a heterozygote individual where the overdominant fitness advantage could be expressed and, therefore, the smaller is the allele advantage. Consequently, balancing selection is less intense as selfing increases [55,59], which makes drift more relevant and increases the rate of fixation for overdominant loci. In fact, for asymmetrical overdominance, the condition for stable polymorphism is *F* < *s*_A_/*s*_a_, where *s*_A_ < *s*_a_ [59]. Thus, for sufficiently high selfing, the allele with the smaller homozygous fitness tends to be removed by selection due to the selfing-induced inbreeding, which implies a sort of purging. In addition, the effective population size of selfed populations is decreased with increased selfing rates (e.g. [64, p. 101]). These factors explain why both the inbreeding load (*B*) and the inbreeding depression of selfed progeny (*δ*) decrease for increasing selfing rates in finite populations even considering that the inbreeding load produced by a symmetrical overdominant model cannot be purged.

Abu-Awad & Waller [35] carried out an analysis of the changes in *δ* in partial selfed populations under a model of pseudo-overdominance, assuming a group of many partially recessive deleterious mutations in repulsion phase in a genomic region of low recombination. For a particular scenario simulated, the model would be equivalent to a single locus where the heterozygote advantage (analogous to our *s_o_* value) is 0.45. The simulation results showed that *δ* remained relatively stable across a broad set of selfing rates and decreased at very high selfing rates. These results are in agreement with ours when a large overdominance effect (*s_o_* = 0.2) is considered (figure 1; electronic supplementary material, S5). Abu-Awad & Waller [35] also looked at how pseudo-overdominance affects load dynamics at recessive deleterious loci and vice versa. Because pseudo-overdominance boosts heterozygosity, mildly deleterious recessive mutations enjoy more protection, boosting their frequency. In our study, we also found a higher average frequency of deleterious mutations in the presence of overdominant loci, even for small overdominant effects (tables 2 and 3), in agreement with those observations. Our results assuming chromosome lengths of one Morgan (electronic supplementary material, figure S3), as it is widely found [63], do not differ much from the assumption of free recombination. However, it is expected that for genome regions with tight linkage, overdominant effects will persist longer and pseudo-overdominance will be facilitated.

### (b) Changes in the inbreeding load of panmictic populations

We have also analysed the genetic changes for fitness traits in panmictic populations after a reduction in their effective size, which can be informative regarding the role of overdominance as a source of inbreeding depression. For example, in a dominance model of deleterious mutations, the mean is expected to decline due to inbreeding and then to partially recover due to purging, and the inbreeding load is expected to be quickly depleted during purging besides being eroded by genetic drift and to approach a new mutation-selection drift equilibrium value. Equations quantifying the expected evolution of both mean fitness and inbreeding load under the dominance model are given by García-Dorado [46]. On the contrary, overdominant inbreeding load cannot be purged by selection in panmictic populations, though overdominant alleles can be lost or fixed due to drift, so that the mean fitness is expected to decline by inbreeding with no recovery, and *B* is expected to decline monotonically as inbreeding progresses due only to genetic drift. In agreement with these expectations, our results show that the most conspicuous difference between the D and the D-OD scenarios is that, whereas mean fitness initially declines but partially recovers by genetic purging in the D scenario, it monotonically declines in all cases where overdominant loci are included, at a rate that increases with the contribution of such loci to the inbreeding load. Although data reporting the long-term evolution of mean fitness in small populations are scarce, a recovery in mean fitness has been experimentally observed under appropriate experimental designs [12], consistent with our observations for the dominance model as well as with theoretical predictions. On the other hand, the inbreeding load itself drops under both scenarios, and, although the relative decline is slowed by the inclusion of overdominant alleles, the difference between both scenarios could be difficult to detect empirically. Scenarios where overdominance contributes substantial inbreeding load also imply either high values of the expressed loads (classical model) or situations where most of adaptive success of the population is ascribed to overdominant mutations (alternative model). We discuss next the particular results obtained with each of the overdominance models considered.

### (c) Classical model of overdominance in the panmictic population

This is the model typically used to obtain theoretical predictions, which shows that, in an equilibrium infinite population, the expressed load *L* generated per locus due to overdominance (the segregation load) is expected to be much larger than that generated by deleterious mutations. Thus, the expressed load expected at equilibrium for symmetrical overdominance is half the selection coefficient added over segregating loci (electronic supplementary material, S1), while, in the dominance model, it is just twice the overall mutation rate [55, p. 56]. Furthermore, the expressed overdominant load equals the inbreeding load per locus (electronic supplementary material, S1), so that overdominance may have a large impact on the average fitness of the population even if it contributes an inbreeding load that is not out of the ordinary values observed in nature. For instance, under our simulation conditions, the expected average fitness at generation zero with the dominance model (with initial *B* = 2.34) would be about *e*^–2*U*^ = *e*^–0.4^ = 0.67, which agrees well with the observed simulated value (*W* = 0.69; figure 2A). When overdominance is included, the expected average fitness at equilibrium is reduced by an additional factor about *e*^–*Bo*^, where *B_o_* is the sum of the loads for all segregating overdominant loci. For example, for the D-OD scenario with *s_o_* = 0.04 and 153 overdominant loci segregating at generation zero, the expected inbreeding load is *B_o_* = 153 × *s_o_* / 2 ≈ 3.06, and the expected average fitness is reduced by a factor *e*^–3.06^ = 0.05. This agrees with the general view that the expressed (segregation) load with overdominance can be very large [24,55]. For instance, in the above scenario, if the segregating load would correspond entirely to viability, each pair of individuals should have 40 offspring for 2 to survive considering only overdominance sources of mortality.

After the reduction in population size, the inbreeding load from all sources is slowly eroded by genetic drift, but that due to deleterious alleles is also quite efficiently depleted by genetic purging during the initial phase. Thus, *B* from non-lethal deleterious alleles is reduced by 51% during the first 50 generations and that due to lethal alleles is reduced by 62% the initial value (table 2). However, after generation 50, these inbreeding loads approach equilibrium values corresponding to a new mutation-selection-drift balance. Thus, from generation 50 to 100, *B* drops only by 18% for non-lethal deleterious and 0% for lethal alleles. For overdominant loci, however, *B_o_* is lost due just to genetic drift, which is opposed by balancing selection. Thus, averaging over both D-OD scenarios with the overdominant effects *s_o_* = 0.04, the inbreeding load ascribed to overdominant alleles is reduced by 24% during the first 50 generations and by 18% from generations 50 to 100 (for *s_o_* = 0.02, *B_o_* is reduced by 30 and 29%, respectively). Thus, the reduction of *B* from overdominant loci is roughly linear on the inbreeding coefficient, as expected from drift, though smaller, due to opposing balancing selection. In any case, the overall inbreeding load declines under all scenarios, although at different rates. In fact, as shown in figure 2, despite the relative decline is initially slower but more sustained in the presence of overdominance, the differences observed between scenarios are not dramatic and can be difficult to detect empirically.

However, a main difference between the two scenarios is apparent regarding the changes of mean fitness. For the D scenario, as expected [46], mean fitness shows a small decline in the initial generations because of inbreeding depression, but, by generation 50, it has virtually recovered the initial value due to the elimination of deleterious and lethal alleles by genetic purging, thereafter showing a slight continuous decline due to the accumulation of new deleterious mutations. The total decline in fitness observed under the D scenario over the whole 250 generations was only about 5%. In contrast, under the classical overdominance hypothesis, a progressive deterioration of the depressed trait over generations would be expected as inbreeding progresses and heterozygotes with advantage are lost [11,25,27]. In fact, our results show that after including overdominant loci (D-OD scenarios in table 2) and despite the reduction in inbreeding load, fitness monotonically decreases more than 20% over the same period, which is due to the continuous loss or fixation of overdominant alleles. This confirmed that the inbreeding load due to overdominance could eventually be removed by genetic drift in populations small enough, but cannot be purged, as pointed out before [28,66].

### (d) Alternative model of overdominance in the panmictic population

We considered an alternative model of overdominance for which the fitness of the homozygotes is one and that of the heterozygotes is higher than one. This model showed results similar to those for the classical model regarding *B*, but, when there are many segregating overdominant loci, average viability becomes close to 1, the realized effects are reduced (electronic supplementary material, S1), and *B* represents the latent inbreeding load that could be fully expressed if average viability was reduced by any cause. However, substantial differences were found for mean fitness, both for fecundity and for viability, the latter being capped at a maximum of one. Thus, for this model, mean fitness with overdominance was initially very large for fecundity and close to one for viability and declined continuously over generations, with a continuous increase of the segregation load for viability (figures 3 and 4).

To interpret the differences between the two overdominant models, we need to understand the biological rationale for each. These classical and alternative models can be seen as just different ways to scale fitness effects, where the first one is widely used for the analytical study of the evolution of allele frequencies and of the equilibrium properties of segregating overdominant loci, while the second one is often used to simulate new overdominant mutations. However, the two models are truly different when it comes to biological realism and the impact of overdominance on the evolution of average fitness, as explained in what follows.

Under the classical model, each new overdominant mutation *m* implies a reduction in the fitness of the wild-type homozygote (++). The more parsimonious interpretation of this feature is that each new overdominant allele is incorporated into the population because, in heterozygosis, it confers some adaptation to an environmental change that reduces the fitness of ++. For example, + could be the wild-type allele segregating against the recessive allele *m* causing sickle-cell anaemia, which would be a rare deleterious allele in an environment without malaria. Then, malaria appears in the area, causing a reduction in the ++ fitness, and the mutant allele *m* happens to confer malaria resistance in heterozygosis, leading to overdominance. This allele *m* could be a new mutation or, more likely, a pre-existing rare allele that was previously deleterious. This model of antagonistic pleiotropy may have occurred on some occasions, for example, in situations implying a change in the environment in the evolutionary history of a species [42]. However, for loci showing antagonistic effects on fitness through two different pleiotropic routes, fitness overdominance requires that the favourable effects in both routes are substantially dominant unless their magnitude is very similar (electronic supplementary material, S1). In addition, it seems unlikely that such evolutionary mechanism could account for important inbreeding load as this implies a very large expressed (segregation) load, meaning a dramatic reduction of mean fitness compared to the situation previous to all the environmentally challenging episodes that prompted the incorporation of such overdominant segregating alleles.

On the contrary, under the alternative model, each new overdominant mutation is in fact an adaptive allele that shows a larger advantage in heterozygosis than in homozygosis and contributes to a net increase in adaptation to the environment. This could be a more frequent biological scenario, which would also imply a much lower expressed segregation load for viability, than the classical model. However, our results show that, when such overdominant loci contribute a large inbreeding load for fecundity, they also cause a several-fold increase in fecundity average (figure 4), so that most of the population reproductive potential is explained by segregating adaptive mutations with overdominant effects, which is evolutionary astounding. In addition, when the alternative model is applied to viability, a high latent inbreeding load implies an average viability close to one (figure 3). Then, as almost all individuals survive, neither the deleterious nor the overdominant effects are truly expressed, and natural selection on viability becomes inefficient. Thus, the realized selection coefficients in the alternative model are considerably reduced compared to the theoretical simulated values (electronic supplementary material, S1). Therefore, high latent inbreeding loads for viability ascribed to the alternative overdominant model can imply an overall relaxation of natural selection that is inconsistent with the well-known evolutionary contention of the deleterious load.

In conclusion, our study shows that both the inbreeding load (*B*) and the inbreeding depression of selfed progeny (*δ*) decrease in finite but relatively large populations as the selfing rate increases, as long as the overdominant effects are not very large. This contrasts with the expectations from infinite population theory that would predict an increase with selfing for the *δ* contributed by overdominant loci. For panmictic populations and after a reduction in population size, the differences in the evolution of the inbreeding load for scenarios assuming dominance and those also including overdominance can be difficult to detect. Nevertheless, when overdominance contributes substantial inbreeding load, mean fitness decays continuously, while it is partially recovered by purging under a pure dominance model. The two overdominance models considered have consequences that constrain a large contribution of overdominant loci to the load. The classical model implies a high segregation load and a too low fitness, and the alternative model generates so high mean fitness that implies inefficient selection for viability, or an unreasonable several-fold increase for fecundity.

## Data Availability

Simulation codes and scripts are available at Zenodo [67]. Supplementary material is available online [68].
